# Isolation and Characterization of *Acinetobacter baumannii* Recovered from *Campylobacter* Selective Medium

**DOI:** 10.3389/fmicb.2016.01871

**Published:** 2016-11-18

**Authors:** Dinesh M. Fernando, Izhar U. H. Khan, Rakesh Patidar, David R. Lapen, Guylaine Talbot, Edward Topp, Ayush Kumar

**Affiliations:** ^1^Department of Microbiology, University of Manitoba, Winnipeg, MB, Canada; ^2^Ottawa Research and Development Centre, Agriculture and Agri-Food Canada, Ottawa, ON, Canada; ^3^Sherbrooke Research and Development Centre, Agriculture and Agri-Food Canada, Sherbrooke, QC, Canada; ^4^London Research and Development Centre, Agriculture and Agri-Food Canada, London, ON, Canada; ^5^Department of Medical Microbiology, University of Manitoba, Winnipeg, MB, Canada

**Keywords:** agriculture niche, microaerophilic, antibiotic susceptibility, RND pumps, MRFPA

## Abstract

*Acinetobacter baumannii*, a Gram-negative opportunistic pathogen, is known to cause multidrug resistant infections. This organism has primarily been isolated from clinical environments and its environmental reservoirs remain largely unknown. In the present study, we recovered seven isolates of *A. baumannii* growing under conditions selective for *Campylobacter* spp. (microaerophilic at 42°C and in the presence of antibiotics) from dairy cattle manure storage tank or surface water impacted by livestock effluents. Antibiotic susceptibility tests revealed that all of these isolates were less susceptible to at least two different clinically relevant antibiotics, compared to the type strain *A. baumannii* ATCC17978. Expression of resistance-nodulation-division efflux pumps, an important mechanism of intrinsic resistance in these organisms, was analyzed, and *adeB* was found to be overexpressed in one and *adeJ* was overexpressed in three isolates. Comparison of these isolates using genomic DNA Macro-Restriction Fragment Pattern Analysis (MRFPA) revealed relatively low relatedness among themselves or with some of the clinical isolates from previous studies. This study suggests that *A. baumannii* isolates are capable of growing under selective conditions for *Campylobacter* spp. and that this organism can be present in manure and water.

## Introduction

The *Acinetobacter* genus is comprised of more than 30 species with the majority being found in soil and water but also as commensal strains isolated from human specimens (Visca et al., 2011). *A. baumannii* is considered the clinically most significant member of this genus, primarily because of its ability to cause serious multidrug resistant infections associated with high mortality rates (Poulikakos et al., 2014) worldwide in immunocompromised individuals (Peleg et al., 2008).

While most *Acinetobacter* species have been isolated from various environmental matrices such as soil and water, *A. baumannii* is rarely found outside of hospital settings. Although, early on the environmental reservoirs of *A. baumannii* outside hospitals were unclear (Peleg et al., 2008), it is becoming increasingly evident that *A. baumannii* can be isolated from different environmental niches (Berlau et al., 1999; Huys et al., 2007; Hamouda et al., 2011). Therefore, it is believed that the clinical isolates of *A. baumannii*, even though they have been studied intensively, do not truly represent the diversity of this species and therefore the study of environmental isolates is important to understand the diverse nature of *A. baumannii* (Diancourt et al., 2010). Considering the growing clinical importance of *A. baumannii*, due to increasing resistance to antibiotics concomitant with an rise in the frequency of infections, a better definition of potential environmental reservoirs and to study possible exposure routes is a priority. In this study, we characterized the isolates of *A. baumannii* that were found to be growing in growth medium semi-selective for *Campylobacter* spp. in a separate study objective of which was to investigate prevalence of *Campylobacter* spp. in agricultural niches (Khan et al., 2014). As the source of *A. baumannii* isolates was nonclinical settings and because the *Campylobacter* spp. selective medium contains a number of antibiotics, the aim of this work was to study their antibiotic susceptibility to clinically relevant antibiotics and also to analyze their relatedness to the clinical isolates of *A. baumannii*.

## Materials and methods

### Isolation of *A. baumannii* from water and manure

*A. baumannii* isolates were recovered from two different sources in a surveillance study which was carried out to investigate the prevalence of *Campylobacter* spp. in agriculture settings. Surface water samples were obtained from the South Nation River (SNR) drainage basin in Eastern Ontario, Canada, from May to November 2013. The drainage basin has numerous watersheds that vary in stream order, intensity of livestock and crop production, wildlife habitat, density of rural residents and proximity to small municipalities. Detailed descriptions of the 200 km^2^ experimental area, local climate conditions, sampling strategy, and sampling methodology have been described previously (Lyautey et al., 2007a,b; Wilkes et al., 2009; Lanthier et al., 2011; Marti et al., 2013). In addition, dairy farms within the drainage basin and a dairy cattle manure storage tank from Agassiz, BC, were sampled.

Initially the samples were processed using Modified Preston broth (Oxoid, Lenexa, KS, USA), an enrichment media supplemented with antibiotics [polymyxin B (5000 IU/L which is equivalent to 0.5 mg/L; Kassamali et al., 2015), rifampicin (10 mg/L), trimethoprim (10 mg/L), and amphotericin B, 10 mg/L]. These tubes were incubated at 42°C under microaerophilic conditions (5% O_2_, 85% N_2_, and 10% CO_2_) for 48 h in a Heracell-150i multi-gas incubator (Thermo Scientific, Waltham, MA, USA). Culture isolates were further purified by streaking using a sterile loop on Modified Karmali agar (MKA) (Oxoid) containing selective antibacterial and antifungal agents [cefoperazone (32 mg/L), vancomycin (20 mg/L), and amphotericin B (10 mg/L)] and followed by incubation at 42°C under microaerophilic conditions for 24–48 h. It should be noted that the resistance breakpoint for polymyxin B for *A. baumannii* is 2–4 mg/L and therefore the concentrations of polymyxin B used in the *Campylobacter* spp. growth medium is 4–8 times lower than the breakpoint (Peleg et al., 2008). After initial isolation, we carried out the sequencing of the 16S rRNA gene amplified using the universal oligonucleotide primers and protocol described previously (Bruce et al., 1992). This was done to confirm the isolates as *Campylobacter* spp. and following this step, 11 isolates were presumptively identified as *Acinetobacter* spp. Sequencing reactions were performed with an ABI PRISM 3130XL Genetic Analyzer (Applied Biosystems, Burlington, ON) at the sequencing facility of Agriculture and Agri-Food Canada, Ottawa, ON. Isolates and oligonucleotide primers are listed in Tables 1, 2, respectively. For subsequent culturing of the isolates, Lysogeny Broth (LB) medium (Difco, BD-Canada, Mississauga, ON) was used.

**Table 1:** **List of strains isolated in this study**.

**Strain**	**Sampling date and site**	**Sampling source and location**
*A. baumannii* AB046	09-07-2013—Stream	Water—SNR^*^ basin, Ottawa, ON^#^, Canada
*A. baumannii* AB047	09-07-2013—River	Water—SNR basin, Ottawa, ON, Canada
*A. baumannii* AB048	09-07-2013—River	Water—SNR basin, Ottawa, ON, Canada
*A. baumannii* AB052	20-08-2013—Stream	Water—SNR basin, Ottawa, ON, Canada
*A. baumannii* AB053	20-08-2013—Stream	Water—SNR basin, Ottawa, ON, Canada
*A. baumannii* AB054	21-10-2013—Storage tank	Dairy cattle manure, Agassiz, BC^∧^, Canada
*A. baumannii* AB055	21-10-2013—Storage tank	Dairy cattle manure, Agassiz, BC, Canada

* *South Nation River*;

# *Ontario*;

∧ *British Columbia*.

**Table 2:** **Primers used in this study**.

**Primer**	**Sequence (5′ → 3′)**	**Target (size of amplicon bp)**	**References**
pA-Forward	AGAGTTTGATCCTGGCTCAG	Universal 16S rRNA (~1500)	Bruce et al., 1992
pH-Reverse	AAGGAGGTGATCCAGCCGCA		
16S-Forward	CTGCCTATTAGTGGGGGACA	16S rRNA of *Acinetobacter* spp. (892)	This study
16S-Reverse	AAGGCACCAATCCATCTCTG		
16S_RT_F	ACATCTCACGACACGAGCTG	16S rRNA of *A. baumannii* (150)	Fernando and Kumar, 2012
16S_RT_R	CGTAAGGGCCATGATGACTT		
adeB_RT_F	GGATTATGGCGACTGAAGGA	adeB^*^ (106)	Fernando and Kumar, 2012
adeB_RT_R	AATACTGCCGCCAATACCAG		
adeG_RT_F	ATCGCGTAGTCACCAGAACC	adeG^*^ (92)	Fernando and Kumar, 2012
adeG_RT_R	CGTAACTATGCGGTGCTCAA		
adeJ_RT_F	CATCGGCTGAAACAGTTGAA	adeJ^*^ (109)	Fernando and Kumar, 2012
adeJ_RT_R	GCCTGACCATTACCAGCACT		

* *adeB, adeG, and adeJ encode the RND-protein in the adeABC, adeFGH, and adeIJK operons, respectively*.

### Identification of *acinetobacter* isolates

Presumptively identified *Acinetobacter* spp. isolates were further analyzed by PCR using the *Acinetobacter* genus-specific 16S rRNA gene primers that amplified a PCR product of 892 bp (Table 2). Regions of 833 bp in length from each of the PCR products were sequenced and compared by multiple sequence alignment and phylogenetic analysis using MegAlign 1993–2012 v10.1.0 (DNASTAR Inc., Madison, WI, USA). Multiple sequence alignments and tree construction were carried out using Clustal-V and Clustal-W pairwise and neighbor-joining methods, respectively. Bootstrapping was performed by creating 1000 trials. The 16S rRNA gene sequences of the seven *A. baumannii* and four *A. indicus* isolates have been deposited to GenBank under accession numbers: AB046, KX955259; AB047, KX955260; AB048, KX955261; AB052, KX955262; AB053, KX955263; AB054, KX955264; AB055, KX955265; AB045, KX955266; AB049, KX955267; AB050, KX955268; and AB051, KX95526.

### Antibiotic susceptibility assays

Minimum inhibitory concentrations (MIC) determination for all antibiotics were carried out using Etest® strips according to manufacturer's protocols (Biomérieux, Montréal, QC, Canada). The panel of antibiotics was chosen based on the ones that are commonly used for the treatment of *A. baumannii* infections (Fernando et al., 2013). Briefly, 3 mL of LB broth was inoculated using a single colony for each strain and incubated at 37°C for 16–18 h with shaking. Cultures were standardized using the 0.5 McFarland standard in saline (0.85% NaCl) and spread on MHB agar (Difco) plates using sterile cotton swabs. Two *E*-test strips of different antibiotics were then placed in opposite orientation onto the inoculated plates and incubated at 37°C for 18 h. The MIC was recorded as the concentration of antibiotics at which the zone of inhibition intersected the *E*-test strip. These assays were done for two biological replicates. Guidelines provided by the Clinical and Laboratory Standards Institute (CLSI) were used to determine if the isolates were resistant, intermediate, or susceptible to the antibiotics (Clinical and Laboratory Standards Institute, 2014).

### RNA extraction and expression of RND pump-encoding genes

Overnight cultures (grown at 37°C with shaking at 250 rpm) were subcultured into 3 mL of LB (1:100) and grown to an absorbance of 0.6–0.8 at 600 nm. One milliliter of this culture was centrifuged, and the cell pellet frozen at −80°C for 1 h to facilitate cell lysis. RNA extraction was performed using the RNeasy kit (Qiagen, Mississauga, ON, Canada) following the manufacturer's instructions. To remove any genomic DNA carryover, the samples were treated with DNase I (Qiagen) for 30 min at 37°C, followed by heat inactivation at 65°C for 5 min. One microgram of total RNA was used to synthesize cDNA using the Bio-Rad iScript reverse transcriptase kit (Bio-Rad, Mississauga, ON, Canada) following the manufacturer's instructions. The control reaction (no reverse transcriptase, NRT) was setup using all components of the reaction but without the reverse transcriptase enzyme. Analysis was carried out on three RND pump-encoding genes (*adeB, adeG*, and *adeJ*) that are a part of *adeABC* (Marchand et al., 2004), *adeFGH* (Coyne et al., 2010), and *adeIJK* operons (Damier-Piolle et al., 2008), respectively. The primers used for qRT-PCR analysis have been described previously (Fernando and Kumar, 2012) and are listed in Table 2. The expression of genes was determined by quantitative PCR using the SsoFast Evagreen Supermix (Bio-Rad) with an Applied Biosystems StepOnePlus™ instrument (Fisher Scientific, Ottawa, ON, Canada). The amplification cycle was as follows: initial denaturation at 95°C for 3 min followed by 40 cycles of 95°C for 10 s and 60°C for 30 s. Two different control reactions were included in the analysis, a no-template control (NTC) and an NRT control. We used the 16S rRNA as the housekeeping control gene to normalize cDNA in all strains. Reactions were set up using a final concentration of 300 nM primers and 2.5 μL of the cDNA template (diluted 1:10) in an 8 μL total reaction volume. All reactions were carried out in triplicate with at least two independent biological replicates. Target gene expression was measured using expression relative to that of *A. baumannii* ATCC17978, for which the expression levels of each gene were set at one. Data analysis was carried out using the Pfaffl method for relative expression (Pfaffl, 2001) and the student *t*-test was used to determine statistical significance and a *P*-value cut off of <0.05 was used to deem data as significant.

### Genotyping using genomic DNA macro-restriction fragment pattern analysis (MRFPA)

Pulsed-field gel electrophoresis (PFGE) was applied to determine the genomic DNA fingerprints of each of the *A. baumannii* isolate using *Apa*I restriction enzyme. The preparation and digestion of the intact genomic DNA and its gel separation by PFGE was carried out according to method described previously (Durmaz et al., 2009). The MRFPA results were interpreted by visual comparison of the bands using previously described criteria (Tenover et al., 1997), whereas the overall interpretation of the restriction patterns was based on both the visual analysis and a dendrogram analysis using the GelCompar-II program (Applied Maths BVBA, Kortrijk, Belgium).

## Results

### Isolation and identification of *acinetobacter* isolates

**Figure 1:**
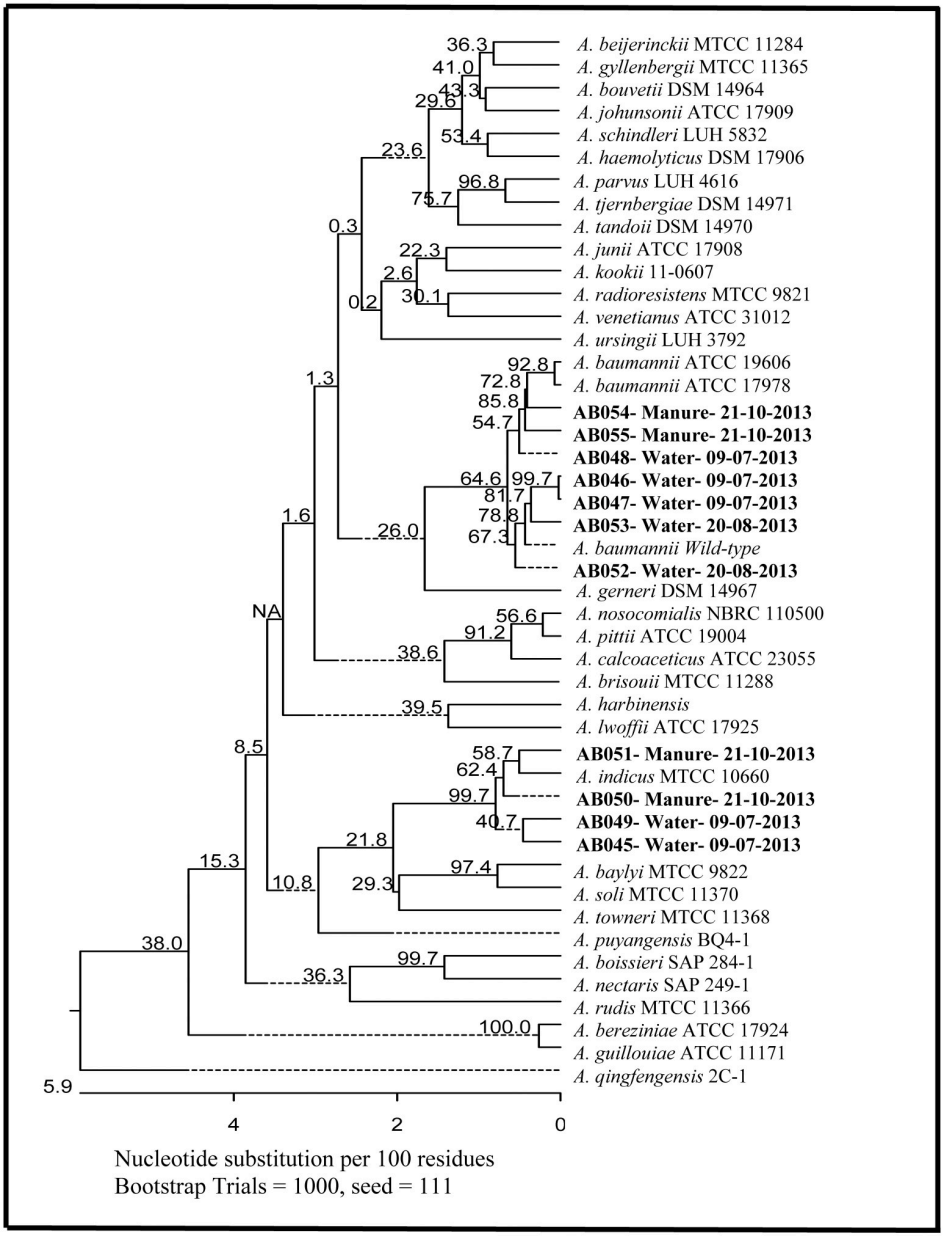
**Phylogenetic tree based on gene encoding 16S rRNA sequences (833 bp) showing the phylogenetic similarity of *Acinetobacter* strains isolated from agricultural water and dairy cattle manure samples with other *Acinetobacter* spp. Bootstrap values based on 1000 replications are shown at the nodes of the tree**. The scale bar represents 5.9% nucleotide sequence divergence.

We recovered 85 isolates from SNR and 256 from the dairy cattle manure storage tank growing under selective conditions for *Campylobacter* spp. (i.e., microaerophilic at 42°C on selective growth medium). However, to our surprise, sequencing of the first set of PCR products from the universal primers for the 16S rRNA gene presumptively identified 11 isolates as *Acinetobacter* spp., seven of which were from SNR and four from dairy cattle manure samples. In order to confirm the identity of these isolates, we carried out *Acinetobacter* genus-specific amplification and sequencing and were able to identify seven of these isolates (AB046, AB047, AB048, AB052, AB053, AB054, and AB055) as *A. baumannii* (Figure 1) and four (AB045, AB049, AB050, AB051) as *A. indicus*. *A. baumannii* AB054 and AB055 and *A. indicus* AB050 and AB051 are from dairy cattle manure while the rest are from SNR water samples. The *A. baumannii* culture isolates were further confirmed to species-level using species-specific PCR assay. As *Acinetobacter* spp. are known to be strict aerobes, we regrew these isolates in pure culture under microaerophilic conditions (5% O_2_, 85% N_2_, and 10% CO_2_) at 42°C along with ATCC17978 as well as three clinical isolates (AB004, AB005, AB006) previously described by us (Fernando et al., 2013). Intriguingly, all of the isolates grew very well under these conditions (data not shown). The seven isolates of *A. baumannii* were further characterized for antibiotic susceptibility, intrinsic resistance as well as for their phylogenetic relationship to some of the clinical isolates.

### Antibiotic susceptibility of *A. baumannii* isolates

Results of antibiotic susceptibility are summarized in Table 3. While none of the isolates were resistant to any of the antibiotics tested, we did find that a number of these display reduced sensitivity, which in some instances was small but reproducible, to antibiotics in comparison to the reference strain *A. baumannii* ATCC17978. Of note are susceptibilities to tigecycline and doxycycline, where all but one isolate (AB054) displayed reduced susceptibility to tigecycline while all isolates were less susceptible to doxycycline when compared to *A. baumannii* ATCC17978. Lower susceptibility to piperacillin/tazobactam was observed in four (AB046, AB047, AB048, and AB053) and to moxifloxacin in four (AB047, AB048, AB053, and AB054) isolates. We also found that AB047 (tigecycline, chloramphenicol, ceftaroline fosamil, doripenem, moxifloxacin, doxycycline, piperacillin/tazobactam), and AB053 (tigecycline, ceftaroline fosamil, cefepime, imipenem, moxifloxacin, doxycycline, piperacillin/tazobactam) displayed reduced susceptibilities to seven antibiotics each and AB046 (tigecycline, imipenem, doripenem, doxycycline, piperacillin/tazobactam) and AB048 (tigecycline, ceftaroline fosamil, moxifloxacin, doxycycline, piperacillin/tazobactam) were less susceptible to five antibiotics each.

**Table 3:** **Antibiotic susceptibility of *A. baumannii* isolates as determined by *E*-test (mg/L)**.

	**TGC**	**AMC**	**CHL**	**CPT**	**FEP**	**IPM**	**DOR**	**MXF**	**DC**	**SXT**	**CLI**	**TZP**
*A. baumannii* AB046	**0.25**	6	0.125	0.75	2	**0.38**	**0.25**	0.047	**0.5**	0.38	0.125	**0.032**
*A. baumannii* AB047	**0.25**	16	**0.25**	**1.5**	2	0.25	**0.25**	**0.064**	**1**	0.19	0.125	**8**
*A. baumannii* AB048	**0.38**	0.5	0.125	**2**	2	0.25	0.125	**0.064**	**1**	0.25	0.38	**8**
*A. baumannii* AB052	**0.25**	4	0.125	0.75	1.5	0.25	0.125	0.032	**0.5**	0.19	0.125	<0.016
*A. baumannii* AB053	**0.38**	16	0.19	**2**	**3**	**0.38**	0.19	**0.064**	**0.75**	0.19	0.38	**0.094**
*A. baumannii* AB054	0.19	0.094	0.125	0.75	0.5	0.094	0.032	**0.064**	**2**	0.125	0.016	<0.016
*A. baumannii* AB055	**0.5**	0.25	0.094	0.38	1	0.19	0.064	0.032	**4**	0.125	0.19	<0.016
*A. baumannii* ATCC 17978	0.19	16	0.19	1	2	0.25	0.19	0.047	0.38	12	0.5	<0.016

Minimum inhibitory concentrations higher than the control strain for each antibiotic are indicated in bold.

TGC, tigecycline; AMC, amoxicillin/clavulanic acid; CHL, chloramphenicol; CPT, ceftaroline fosamil; FEP, cefepime; IPM, imipenem; DOR, doripenem; MXF, moxifloxacin; DC, doxycycline; SXT, trimethoprim/sulfamethoxazole; CLI, colistin; TZP, piperacillin/tazobactam.

### Expression of RND efflux pumps in *A. baumannii* isolates

**Figure 2:**
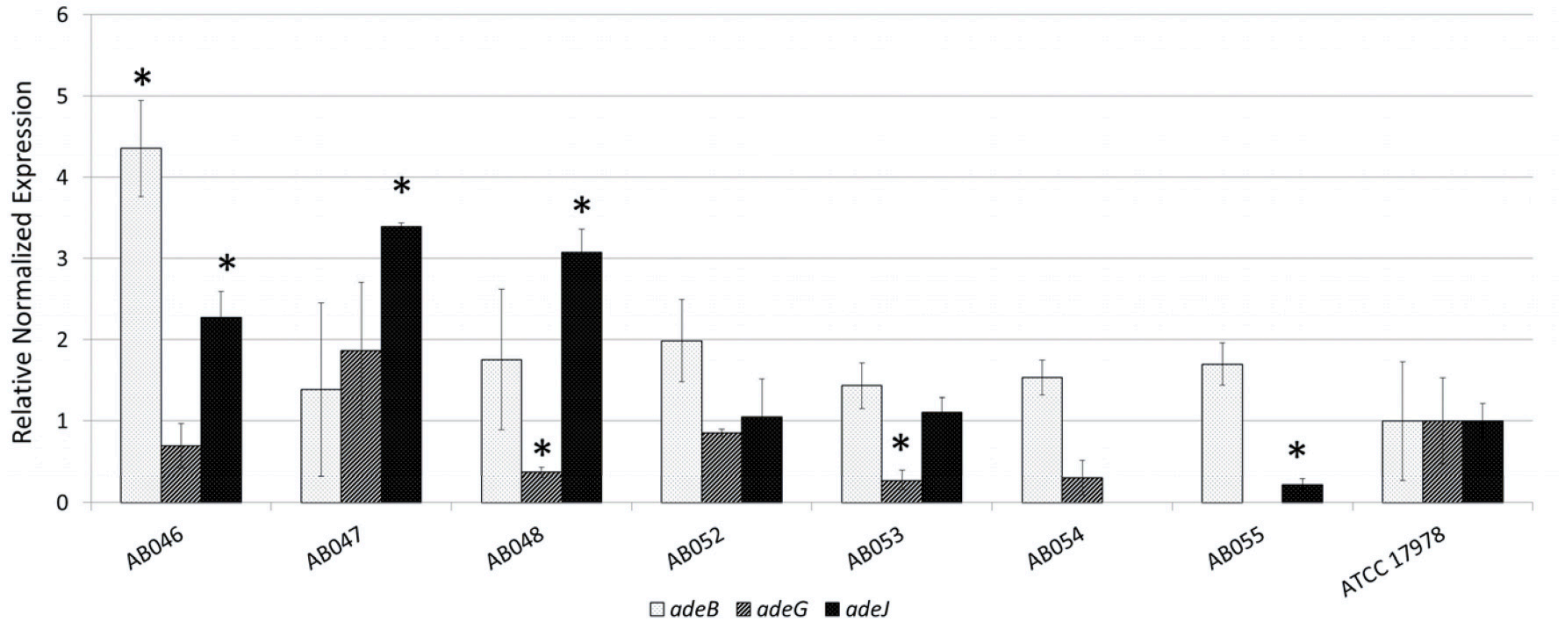
**Relative expression of RND efflux pump genes (*adeB*, *adeG*, *adeJ*) in environmental isolates of *A. baumannii***. Values were measured relative to expression of genes in ATCC 17978 (set to one for each) using 16S rRNA gene as the housekeeping control. Error bars represent standard deviation. Significant changes in expression (*p* < 0.05) relative to ATCC 17978 are indicated by an asterisk.

Results of the qRT-PCR analyzing the expression of RND pump-encoding genes are shown in Figure 2. The Ct values for the housekeeping 16S rRNA gene for all seven test strains as well as the control ATCC17978 was within one cycle, therefore validating our choice of 16S rRNA as a housekeeping control gene. We detected the *adeB* transcript in all strains, although its overexpression (with respect to the control strain ATCC17978) was observed in only one isolate, AB046. We also observed *adeG* transcript in all but one isolate (AB055), however the expression of *adeG* was either similar to or lower than the wild-type control in isolates in which the transcript was detected. *adeJ* was the most commonly overexpressed gene, with overexpression observed in three isolates (AB046, AB047, and AB048). AB054 was the only isolate in which we did not detect the *adeJ* transcript and incidentally was the most susceptible to tigecyline.

### Macro-restriction fragment pattern analysis

**Figure 3:**
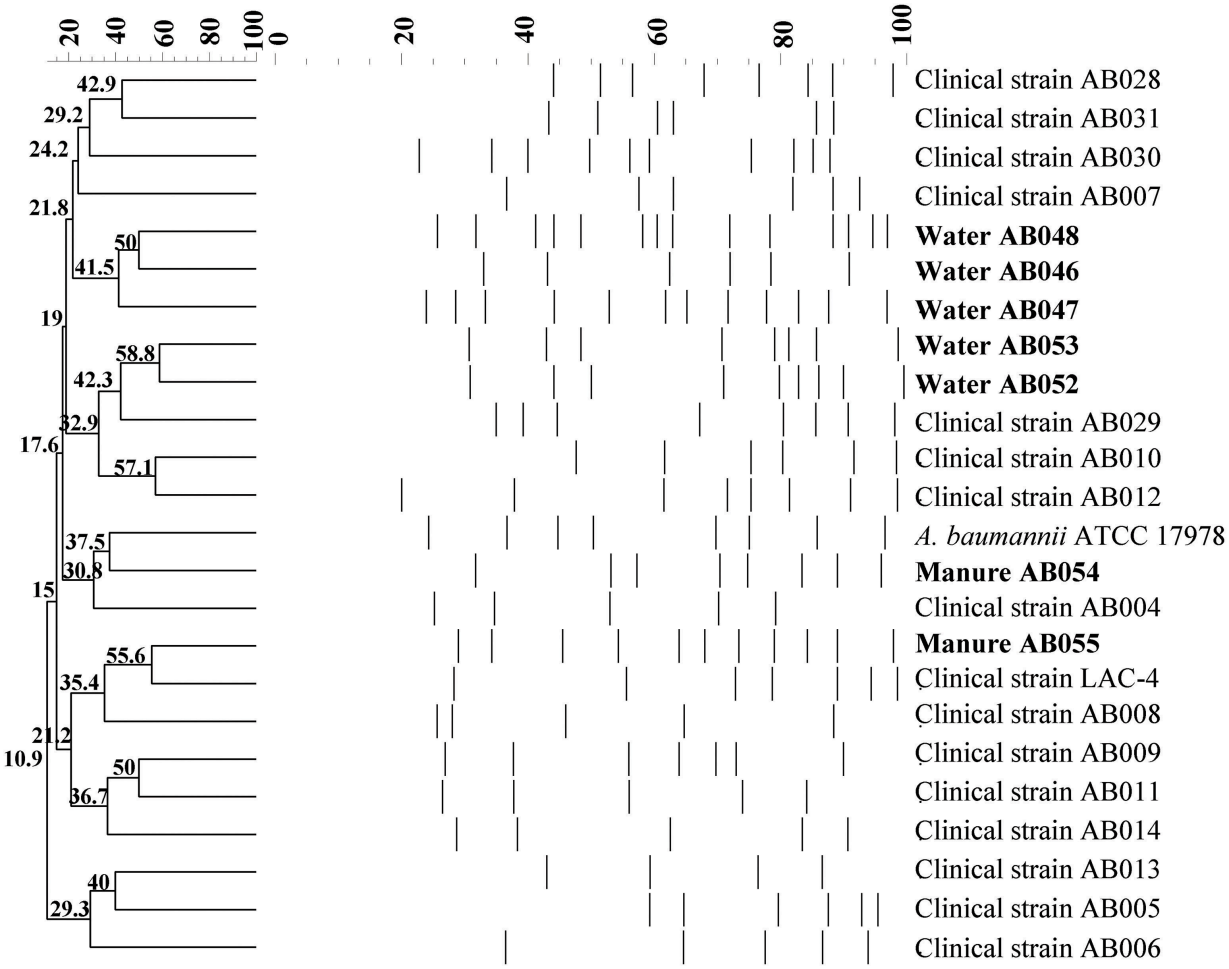
**Dendrogram analysis of *Apa*I PFGE patterns of chromosomal DNA of *A. baumannii* isolates where degree of similarity of macrorestriction patterns of 24 *A. baumannii* reference, environmental, and clinical strains are shown**.

The seven isolates were further characterized for strain-level variation and compared with 17 clinical isolates using PFGE. Digestion with *Apa*I restriction enzyme produced 6–14 readable and distinctive MRFPA bands for the different isolates. To determine the degree of similarity of the different PFGE patterns, the 24 tested strains were examined by dendrogram analysis. The clustering of the PFGE types and the origin of isolates confirmed the non-identical status of the different strains (Figure 3). The dendrogram results displayed 24 unique DNA patterns. An overall 30% pattern similarity was observed which showed a high degree of genetic diversity among environmental and clinical strains representing the isolates originating from different geographic sources.

## Discussion

In this study, while screening for *Campylobacter* spp. from agricultural niches, we recovered *A. baumannii* isolates growing on growth medium selective for *Campylobacter* spp. These isolates were confirmed as *A. baumannii* using 16S rRNA gene sequencing which was further corroborated by successful amplification reactions for efflux pumps genes, which are specific PCR assays for *A. baumannii*. We went on to further characterize these isolates for two primary reasons. One, because *A. baumannii* is typically not found in the environment (Peleg et al., 2008) and most of the isolates studied to date are of clinical origin (Apisarnthanarak and Mundy, 2009; Adams et al., 2010; Fernando et al., 2013), study of non-clinical isolates is vital as it can provide important insights into how the organism may have evolved into a multidrug resistant bacterium. Second, as the selective growth medium for *Campylobacter* spp. used in our study contains a number of antimicrobials (polymyxin B, rifampicin, trimethoprim, amphotericin, cefoperazone, and vancomycin), we were interested in studying the antibiotic susceptibility of these isolates to clinically relevant antibiotics. In particular, growth of these isolates on medium containing polymyxin B of interest, as it is the “last-resort” antibiotic for multidrug resistant *A. baumannii* infections. Intriguingly, these isolates were found to be growing under microaerophilic conditions that are not considered suitable for *Acinetobacter* spp. as they are known to be strict aerobes. However, we were able to culture not only the environmental isolates from this study but also the type strain ATCC17978 as well as clinical isolates previously described by us (Fernando et al., 2013), with the growth rate similar to that under aerobic conditions (data not shown). This shows that at least some strains of *A. baumannii* including the type strain ATCC17978 are able to grow under these conditions.

We analyzed the *A. baumannii* isolates for their resistance to clinically relevant antibiotics as well as for the expression of RND pumps, major contributors of intrinsic resistance of organisms like *A. baumannii* (Bonomo and Szabo, 2006; Li et al., 2015). Although none of the isolates were resistant to any of the antibiotics by clinical definition (Clinical and Laboratory Standards Institute, 2014), a number of these were less susceptible to these antibiotics than the reference strain *A. baumannii* ATCC17978 (Table 3). Analysis of the expression of three characterized RND pumps of *A. baumannii*, AdeABC, AdeFGH, and AdeIJK in our isolates was carried out by measuring the expression of RND pump-encoding genes (*adeA, adeG*, and *adeJ*) in the respective operons (Figure 2). These pumps have been well characterized in clinical isolates of *A. baumannii* from various sources (Chu et al., 2006; Coyne et al., 2009; Fernando et al., 2013). While we did find some isolates (AB046, AB047, and AB048) that overexpressed one or more of these pumps relative to *A. baumannii* ATCC17978, we did not find a clear correlation between reduced susceptibility to antibiotics (Table 3) and overexpression of pumps (Figure 2). For example, we observed that majority of isolates were less susceptible to tigecycline and doxycycline compared to *A. baumannii* ATCC17978 (Table 3). Tigecycline is the drug of choice for pandrug resistant *A. baumannii* infections (Doi et al., 2015). On the other hand, reduced susceptibility to doxycycline is not uncommon in clinical isolates of *A. baumannii* (Castanheira et al., 2014). Resistance to tigecycline in *A. baumannii* often results from the activity of AdeABC and AdeIJK efflux pumps (Peleg et al., 2007; Potron et al., 2015), while doxycycline resistance can result from the overexpression of AdeIJK pump (Fernando et al., 2014). However, we did not see a correlation between reduced susceptibility to tigecycline and doxycycline and the overexpression of AdeABC or AdeIJK pump. Still, we did observe that isolates that displayed higher expression of pumps (AB046, AB047, and AB048) were also less susceptible to a larger number of antibiotics compared to the wild-type control (Table 3). However, this lack of correlation between pump expression and antibiotic susceptibility is not entirely surprising as a clear correlation between the expression of efflux pumps and antibiotic susceptibility in clinical/environmental isolates is sometimes difficult to establish, as shown previously in various organisms (Everett et al., 1996; Chuanchuen et al., 2008; O'Regan et al., 2008) including *A. baumannii* (Fernando et al., 2013). This is most likely due the fact that efflux is not the only mechanism responsible for reduced antibiotic susceptibility of such isolates. With respect to the pump expression in these isolates, we observed one curious difference from the clinical isolates that we characterized previously (Fernando et al., 2013) where *adeG* was found to be the most commonly overexpressed RND pump gene whereas in the environmental isolates described in this study, *adeG* expression was lower than the control strain for all tested strains.

All of the isolates in the current study were susceptible to antibiotics which is akin to what we have observed for some of the clinical isolates of *A. baumannii* from Canadian hospitals, previously (McCracken et al., 2009; Fernando et al., 2013). We determined the degree of relatedness of the isolates from the current study to the clinical isolates of *Acinetobacter* spp. by genomic DNA fingerprinting using PFGE. This technique has been used to study epidemiological relationship and monitor inter-institutional, regional, and international transmission of multidrug-resistant *Acinetobacter* species (Nemec et al., 2004; Cornaglia et al., 2007; van Belkum et al., 2007). Overall there was very low degree of relatedness (30%) amongst strains (Figure 3). Low degree of relatedness has previously been reported for clinical isolates of *A. baumannii* from Canadian hospitals as well (McCracken et al., 2009). In summary, based on the MRFPA, antimicrobial susceptibility, as well as the expression of efflux pumps, we conclude that the isolates in this study are quite diverse from any of the clinical isolates of *A. baumannii* previously described by us (Fernando et al., 2013) as well as others (Golanbar et al., 2011).

To the best of our knowledge, this is the first study where *A. baumannii* has been recovered from environmental conditions that are selective for *Campylobacter* spp. This study further highlights the versatility of this important organism in its ability to grow under various environmental conditions and thus underscores its ability to grow on *Campylobacter* selective medium. Our work also shows that the isolates of *A. baumannii* can be extremely diverse. While none of the strains displayed resistance to antibiotics as per the clinical definition, some of these isolates display lower antibiotic susceptibility compared to the type strain and therefore in future they can serve as an important resource to study the development of antibiotic resistance in *A. baumannii*.

## Author contributions

AK, IK, ET, GT, and DL designed the experiments. DF and IK carried out the experiments. AK, DF, IK, ET, GT, and DL analyzed the data and wrote the manuscript.

## Funding

This work was supported by a Discovery Grant (2015-05550) from the Natural Science and Engineering Research Council of Canada (NSERC) (AK) and the Growing Forward 2 program (Project #'s: 1074 and 1236) of Agriculture and Agri-Food Canada (AAFC) (IK). DF is supported by a Canada Research Chair (CRC) program grant to Peter C. Loewen, University of Manitoba.

### Conflict of interest statement

The authors declare that the research was conducted in the absence of any commercial or financial relationships that could be construed as a potential conflict of interest.
